# Medical prescribing and antibiotic resistance: A game-theoretic analysis of a potentially catastrophic social dilemma

**DOI:** 10.1371/journal.pone.0215480

**Published:** 2019-04-19

**Authors:** Andrew M. Colman, Eva M. Krockow, Edmund Chattoe-Brown, Carolyn Tarrant

**Affiliations:** 1 Department of Neuroscience, Psychology and Behaviour, University of Leicester, Leicester, United Kingdom; 2 Department of Health Sciences, University of Leicester, Leicester, United Kingdom; 3 Department of Media, Communication and Sociology, University of Leicester, Leicester, United Kingdom; Beijing Normal University, CHINA

## Abstract

The availability of antibiotics presents medical practitioners with a prescribing dilemma. On the one hand, antibiotics provide a safe and effective treatment option for patients with bacterial infections, but at a population level, over-prescription reduces their effectiveness by facilitating the evolution of bacteria that are resistant to antibiotic medication. A game-theoretic investigation, including analysis of equilibrium strategies, evolutionarily stability, and replicator dynamics, reveals that rational doctors, motivated to attain the best outcomes for their own patients, will prescribe antibiotics irrespective of the level of antibiotic resistance in the population and the behavior of other doctors, although they would achieve better long-term outcomes if their prescribing were more restrained. Ever-increasing antibiotic resistance may therefore be inevitable unless some means are found of modifying the payoffs of this potentially catastrophic social dilemma.

## Introduction

Antibiotic medication is the treatment of choice for patients who present with symptoms consistent with bacterial infection, but it is difficult for a doctor to be certain that the treatment is appropriate for the condition, because the symptoms typically have other possible causes. Broad-spectrum antibiotics, given their effectiveness against a wide range of bacteria, provide especially useful treatments that can be begun even before specific pathogens have been identified in symptomatic patients. By enabling rapid commencement of treatment, they reduce the risk of serious morbidity and mortality—illness and death in the population. However, whenever antibiotics are prescribed, they facilitate the evolution and spread of antibiotic-resistant bacteria, and antibiotic over-prescription accelerates this process. Antibiotic resistance could lead to the emergence of communicable diseases impervious to all currently available medications, and this may ultimately trigger destructive pandemics. At a special meeting of the United Nations in 2016, the President of the 71st session of the General Assembly declared that antimicrobial resistance has the capacity to kill millions of people each year and that “ultimately, the future of humanity may depend on our ability to respond to the great challenges of antimicrobial resistance” [1].

Several mathematical models of antimicrobial resistance have been published—for reviews, see [2–4]. Some previous research has even focused on game-theoretic models of the evolution of resistance at the level of competing bacteria [5], but we are unaware of any game-theoretic models of the antimicrobial prescribing behavior of medical practitioners or physicians. Game theory is a branch of mathematics devoted to the analysis of interactive decision making, and game-theoretic models provide an appropriate conceptual framework for the analysis of antimicrobial prescribing decisions. A recent survey [6] revealed that 96% of a sample of 1,530 infectious disease professionals in the US believed that antibiotic over-prescription could result in a *tragedy of the commons*—a social dilemma in which rational decision making leads to a collective outcome that is suboptimal for all. A modeling study [7] provided support for this assertion by demonstrating that a conflict of interest between the individual and the community may indeed arise in some relevant circumstances, but this study did not aim to model the prescribing behavior of doctors. Although several authors have suggested that antibiotic over-prescribing can be understood as a tragedy of the commons, the claim has not been formally proved, and the primary aim of this article is to fill this gap in the literature.

In the sections that follow, we use techniques of orthodox game theory and evolutionary game theory to investigate the strategic structure of the problem. We begin by formalizing an antibiotic prescribing population game, and we establish that the game has a unique Nash equilibrium. A limitation of equilibrium analysis is that it reveals what strategies rational decision makers will choose if all others are also behaving rationally—by maximizing utility according to the assumptions of game theory—but it does not reveal whether or not an evolutionary process could lead to other strategies replacing these existing strategies when the game is repeated indefinitely. To examine the robustness of the Nash equilibrium to replacement by other strategies, we show that the Nash equilibrium is an evolutionary stable strategy (ESS) and is therefore invulnerable to replacement by evolutionary change.

Nash equilibrium and ESS are both static solution concepts: they describe what occurs when all or most players adopt the component strategies of an equilibrium or an ESS, but they provide no insight into how equilibrium states can be attained by incremental changes over time, or more generally what happens when behavior deviates radically from equilibrium. Von Neumann and Morgenstern [8] recognized this limitation of the equilibrium analysis that they developed: “We repeat most emphatically that our theory is thoroughly static. A dynamic theory would unquestionably be more complete and preferable” (p. 44). We therefore complete our analysis by examining this question using replicator dynamics, a technique designed to answer precisely these questions. The mathematical techniques of evolutionary stability and replicator dynamics were originally designed to explain biological evolution through natural selection; but it has been generally acknowledged since the 1990s that they are equally applicable to cultural or memetic evolution that occurs through adaptive learning in repeated games, such as the one being described here [9].

### Antibiotic prescribing game

#### Specification of the game

We begin by specifying a symmetric multiplayer population game

G≔〈N,S,π〉,

where *N* = {1, 2, …, *n*} is a set of players comprising all medical practitioners authorized to prescribe antibiotic medication within a well-defined population, *S* is a common strategy set for these players, and $\pi :S^{n}\to {\mathbb{R}}^{n}$ is their common expected payoff or utility function. We assume that *n* ≥ 2 and that *S* contains a choice of exactly two pure strategies that we label T (treat symptomatic patients with antibiotics) and U (do not treat symptomatic patients with antibiotics). A mixed strategy for Player *i* (*i* ∈ *N*) can therefore be written simply as the probability *p*_*i*_∈*P* with which *i* chooses T, where *P* = [0, 1]. Players make repeated decisions—this is an indefinitely repeated game. We use the symbol *q* for the proportion of symptomatic patients in the population who are currently medicated with antibiotics.

We call **p** = (*p*_1_,*p*_2_,…,*p*_*n*_),*p*_*i*_∈*P*, a *strategy profile*. To denote the *incomplete strategy profile* excluding Player *i*, we write

$$p_{-i}=\left(p_{1},\;p_{2},\ldots ,\;p_{i-1},\;p_{i+1},\ldots ,\;p_{n}\right)\in \prod_{j\neq i}P_{j},$$

and we extend this notation by writing

$$\pi_{i}\left(p_{i},\;p_{-i}\right)=\pi \left(p_{1},\;p_{2},\ldots ,\;p_{i-1},\;p_{i},\;p_{i+1},\;\ldots ,p_{n}\right)$$

to denote the payoff to a Player *i* who chooses a specified strategy *p*_*i*_ while the remaining players choose strategies *p*_*j*≠*i*_.

### Nash equilibrium

In a Nash equilibrium, by definition,

$$\pi_{i}\left(p_{i}^{*},p_{-i}^{*}\right)\geq \pi_{i}\left(p_{i},p_{-i}^{*}\right)$$ (1)

for all *p*_*i*_∈*P* and for all *i* ∈ *N* [10, 11]. If Inequality 1 holds strictly, then $p_{i}^{*}$ is also an *evolutionarily stable strategy*, a concept introduced by Maynard Smith and Price [12] and developed by Maynard Smith [13, 14]. On the other hand, if for some *p*_*i*_∈*P*, $\pi_{i}\left(p_{i}^{*},p_{-i}^{*}\right)=\pi_{i}\left(p_{i},p_{-i}^{*}\right)$ and

$$\pi_{i}\left(p_{i}^{*},p_{-i}\right)>\pi_{i}\left(p_{i},p_{-i}\right),$$ (2)

for all *p*_*i*_∈*P* and for all *i* ∈ *N*, then $p_{i}^{*}$ is once again an evolutionarily stable strategy. Thus, an evolutionarily stable strategy $p_{i}^{*}$ is either a strict, symmetric, Nash equilibrium or, if Inequality 1 holds with equality, it is a symmetric Nash equilibrium in which $p_{i}^{*}$ yields a higher payoff against any other mixed strategy than that other mixed strategy yields against itself. Taken together, Inequalities 1 and 2 imply

$$\pi_{i}\left(p_{i}^{*},\epsilon p_{-i}+\left(1-\epsilon \right)p_{-i}^{*}\right)>\pi_{i}\left(p_{i},\epsilon p_{-i}+\left(1-\epsilon \right)p_{-i}^{*}\right)$$ (3)

for all **p**_−*i*_ and for all *i* ∈ *N*, where ϵ > 0 is sufficiently small proportion of the population adopting a non-Nash strategy (to be precise, ϵ is sufficiently small if for all *p*_*i*_∈*P* there exists *δ* > 0 such that Inequality 3 holds for every ϵ satisfying 0 < ϵ < *δ*).

Eq 3 provides an intuitive interpretation of evolutionary stability. Not only do Inequalities 1 and 2 imply 3, but Inequality 3 implies 1 and 2 (see Appendix for a proof).

#### Payoff functions

We assume that doctors make prescribing decisions in the context of patients presenting with signs or symptoms of possible bacterial infection but without conclusive evidence of the underlying pathology. This is a common decision context in both primary and acute care. We assume further that doctors are motivated exclusively to minimize morbidity in their patients, without attaching significant weight to wider and longer-term considerations. These may seem a strong assumptions, but medical students are taught to consider the welfare of their own patients as paramount and to give priority to managing immediate clinical risks [15], and there is compelling evidence that they do indeed tend to focus more on the potentially serious consequences of failing to prescribe when a patient has a bacterial infection than on the consequences of prescribing when a patient is not infected [16–17]. The extent to which some doctors may also be motivated by societal imperatives of antibiotic stewardship, or may see themselves as arbitrators between competing patient and societal demands, are empirical questions that our model will ignore. It is useful to examine the logical implications of purely patient-centered motivations, arising exclusively from the doctors’ interests in the outcomes for their own patients.

Considering a population of prescribing doctors at a particular time, and adapting notation commonly used in the literature on vaccination [18], we specify the morbidity risks to patients—the probability that they will suffer serious illness, possibly including death—both if they are treated and if they are untreated with antibiotics. Because doctors are assumed to be motivated to do the best for their patients, the lower the morbidity among their patients, the higher the doctors’ payoffs. For patients treated with antibiotics, we denote the morbidity rate as *r*^T^, but we usually write it as $r_{q}^{T}$ to indicate that it is dependent on the proportion *q* of patients in the population who are currently treated with antibiotics. It is clear that $r_{q}^{T}$ is dependent on *q*, because increasing *q* causes greater resistance in the community with an associated increasing risk of patients becoming infected by resistant bacteria. As a consequence, the morbidity risk to antibiotic-treated patients will also increase along with the risk that the antibiotics will no longer be effective against their infections. The corresponding morbidity rate for those untreated with antibiotics is *r*^U^, and it is independent of *q*. We use the symbol *ϕ* ∈ (0, 1] for the nonzero probability that a symptomatic patient does indeed have a bacterial infection, because this is typically uncertain when a symptomatic patient presents. Although antibiotic medication can cause side-effects such as diarrhea, these typically amount to nothing more than minor complications. We therefore assume that antibacterial medication has no significant effect on the morbidity of patients who do not have bacterial infections. A final standard simplifying assumption is that all doctors have the same information and respond to it in the same way, seeking to maximize their expected payoffs according to standard assumptions of game theory.

With this notation, a doctor *i*’s expected payoff from choosing the strategy of prescribing antibiotics with probability *p*_*i*_, while the proportion of symptomatic patients currently treated with antibiotics is *q*, is

$$\pi_{i}\left(p_{i},q\right)=p_{i}\left(-r_{q}^{T}\phi \right)+\left(1-p_{i}\right)\left(-r^{U}\phi \right).$$ (4)

In game theory, payoffs are utilities, determined or revealed by the players’ own choices according to the axioms of von Neumann Morgenstern [8], and they are measured on an interval scale; hence the strategic properties of a game are unaffected by dividing all payoffs by a positive constant. Assuming that the morbidity rate in patients untreated with antibiotics is never zero (*r*^U^>0), so that there is some positive probability that they will become ill, we can simplify Eq 4 by dividing through by *r*^U^ and writing it in terms of the relative morbidity risk $r_{q}=r_{q}^{T}/r^{U}$. Thus,

$$\pi_{i}\left(p_{i},q\right)=\phi \left[p_{i}\left(1-r_{q}\right)-1\right].$$ (5)

When *q* = 0, the relative morbidity risk *r*_*q*_ is zero (or at least close to zero), because without antibiotic prescribing there is no antibiotic resistance in the population, and antibiotics are maximally effective. In this case, $r_{q}^{T}=0,$ and therefore $r_{q}^{T}/r^{U}=r_{q}=0.$ As *q* increases, *r*_*q*_ increases, and is reasonable to assume that *r*_*q*_ eventually reaches unity when *q* = 1, because antibiotic resistance can ultimately render antibiotics entirely ineffective, so that, apart from possible side effects of medication, the relative morbidity rates in treated and untreated patients are effectively the same: $r_{q}^{T}=r^{U},$ and consequently $r_{q}^{T}/r^{U}=r_{q}=1.$ The growth of antibiotic resistance is driven by random mutations, the mutation rate in bacteria is remarkably stable at approximately 0.003 mutations per genome per cell generation [19], and the relation between *q* and *r*_*q*_ is therefore approximately linear. The mathematical basis of the linear growth in antibiotic resistance was worked out in 1981 [20] and has subsequently been observed in empirical studies, for example [21–23].

These considerations allow us to simplify further by setting *r*_*q*_ = *q*, and therefore

$$\pi_{i}\left(p_{i},q\right)=\phi \left[p_{i}\left(1-q\right)-1\right].$$ (6)

If we were to assume instead that antibiotic resistance never reduces antibiotic effectiveness to zero, even in the extreme case of *q* = 1, then a modified version of the model would require *r*_*q*_ = *kq*, where *k* is a constant (0 < *k* < 1), but that would not drastically affect the main results of our analysis. Eq 6 is the basic payoff function of the antibiotic prescribing game.

Recall that we label antibiotic treatment decisions T and other treatment decisions U. The payoff is *π*_*i*_(1,*q*) to a doctor *i* choosing T with certainty and *π*_*i*_(0,*q*) to a doctor choosing U with certainty. Setting *p*_*i*_ = 1 and *q* = 1 in Eq 6, when the proportion of patients in the community medicated with antibiotics is 1, the payoff for T is *π*_*i*_(1,1) = −*ϕ*. In the same manner, setting *p*_*i*_ = 1 and *q* = 0, the payoff for T is *π*_*i*_(1,0) = 0.

**Fig 1:**
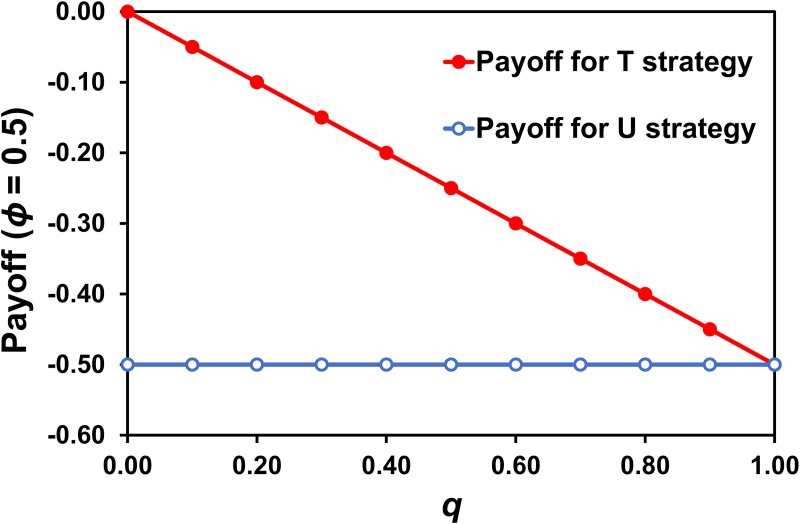
Payoff functions. Payoffs from T (treat with antibiotics) and U (do not treat with antibiotics) for a representative probability of bacterial infection of *ϕ* = .5, showing T as a dominant strategy. For different values of *ϕ* > 0, only the slope of the function changes. The values used to generate the graph are given in S1 Table.

Fig 1 shows the payoffs for the strategies T or *π*_*i*_(1,*q*) and U or *π*_*i*_(0,*q*) for a representative bacterial infection probability of *ϕ* = .5. With different infection probability values *ϕ*, the only changes are the level of the payoff for the U strategy and consequently the slope of the payoff for the T strategy. It is clear that T yields better payoffs than U and that this holds for all values 0 < *q* < 1, and it also holds for all values of 0 < *ϕ* ≤ 1. This means that T is a dominant strategy, as is obvious in Fig 1.

The payoff *π*_*i*_(*p*_*i*_,*q*) is maximized in a population in which every doctor always chooses *p*_*i*_ = 1/2. If we assume that half the symptomatic patients are treated with antibiotics, so that *p*_*i*_ = *q*, and from Eq 6, we have *π*_*i*_(*p*_*i*_,*q*) = *ϕ*[*q*(1−*q*)−1]. This payoff is maximized when *q* = 1/2, for *ϕ* > 0 and 0 < *q* > 1. It follows that, although T is a dominant strategy, every doctor receives a better payoff from choosing any other strategy *p*_*i*_ (0 < *p*_*i*_ < 1), and every doctor receives the best possible payoff if all doctors choose the strategy *p*_*i*_ = 1/2.

## Evolutionarily stable strategy

The payoff to a doctor *i* for the pure strategy T (treat with antibiotics with certainty) can be derived from Eq 6 by setting *p*_*i*_ = 1:

$$\pi_{i}\left(1,q\right)=-\phi q,$$ (7)

and the payoff for the pure strategy U (do not treat with antibiotics) by setting *p*_*i*_ = 0:

$$\pi_{i}\left(0,q\right)=-\phi .$$ (8)

For all values of *q* < 1 and all values of *ϕ* (0 < *ϕ* ≤ 1), we have *π*_*i*_(1,*q*)>*π*_*i*_(0,*q*), confirming that T is a dominant strategy. With the assumptions of our model, the Nash strategy is p* = 1. For all *ϕ* ∈ (0, 1], Inequality 1 is satisfied strictly, hence we can write it in the form

$$\pi_{i}\left(p_{i}^{*},p_{-i}^{*}\right)>\pi_{i}\left(p_{i},p_{-i}^{*}\right),$$

and therefore $p_{i}^{*}$ is also an evolutionarily stable strategy. This means that if most doctors adopt this strategy, and a small proportion of others choose any other strategy *p* ≠ p*, then the majority receive strictly better payoffs, as shown by Inequality 3. It also means that, among the population of doctors as a whole, any perturbation or small deviation from p* will tend to be self-correcting.

### Replicator dynamics

We turn finally to an analysis of how the behavior of doctors in the antibiotic prescribing game evolves over time, as the players make repeated prescribing decisions. Our Nash equilibrium and evolutionary stability analyses have established what occurs when all or most players choose the dominant strategy, but we have not yet discovered what happens if the prescribing behavior of doctors is radically out of equilibrium, nor have we shown whether equilibrium and ESS will arise through incremental changes over time.

Early steps toward a dynamic game theory were made by Nash in a passage in his doctoral dissertation submitted in 1950 [24]. We use the now well-developed mathematical theory of *replicator dynamics*, designed to model the process whereby strategy choices in games change over time. The basic principles of replicator dynamics were introduced by Taylor and Jonker [25], and the theory was developed further by Weibull [26], Hofbauer and Sigmund [27], and Young [28], among others. Replicator dynamics show how the game evolves from all possible initial states. By modeling explicitly the process whereby individual players decide repeatedly whether or not to switch strategies, these and other researchers have shown how replicator dynamics can be used to analyze adaptive learning in games. The process does not converge in all games, but when it does, it converges ultimately to Nash equilibrium.

The replicator equation indicating the increase or decrease of antibiotic treatment decisions T, given the current population relative frequency *q* of T, is defined as the current relative frequency of T multiplied by the payoff for T relative to the average payoff for T and U. The replicator equation relevant to the antibiotic prescribing game can be derived without difficulty (see the Appendix for a proof) and turns out to be

$$\dot{q}=\frac{dq}{dt}=\phi q{\left(1-q\right)}^{2}.$$ (9)

Values of $\dot{q}$ define a vector field, specifying the direction and rate of change in the relative frequency of T choices in the population, positive values indicating increasing T and negative values decreasing T. The rest points in Eq 9 are *ϕ* = 0, *q* = 0, and *q* = 1. For all other values of *q*, and for all values of *ϕ* > 0, $\dot{q}$ is positive, increasing as *ϕ* increases from 0 to 1, and increasing then falling as *q* increases from 0 to 1, indicating increasing rate of increase of T choices. We have assumed that *ϕ* > 0, and this implies that, except when every symptomatic patient within the population has been prescribed antibiotic medication, the decisions to prescribe antibiotics become increasingly frequent until, in equilibrium, all treatment decisions for symptomatic patients involve antibiotics. The role that the infection probability *ϕ* plays is to accelerate the drift to T at a constant rate as *ϕ* increases.

**Fig 2:**
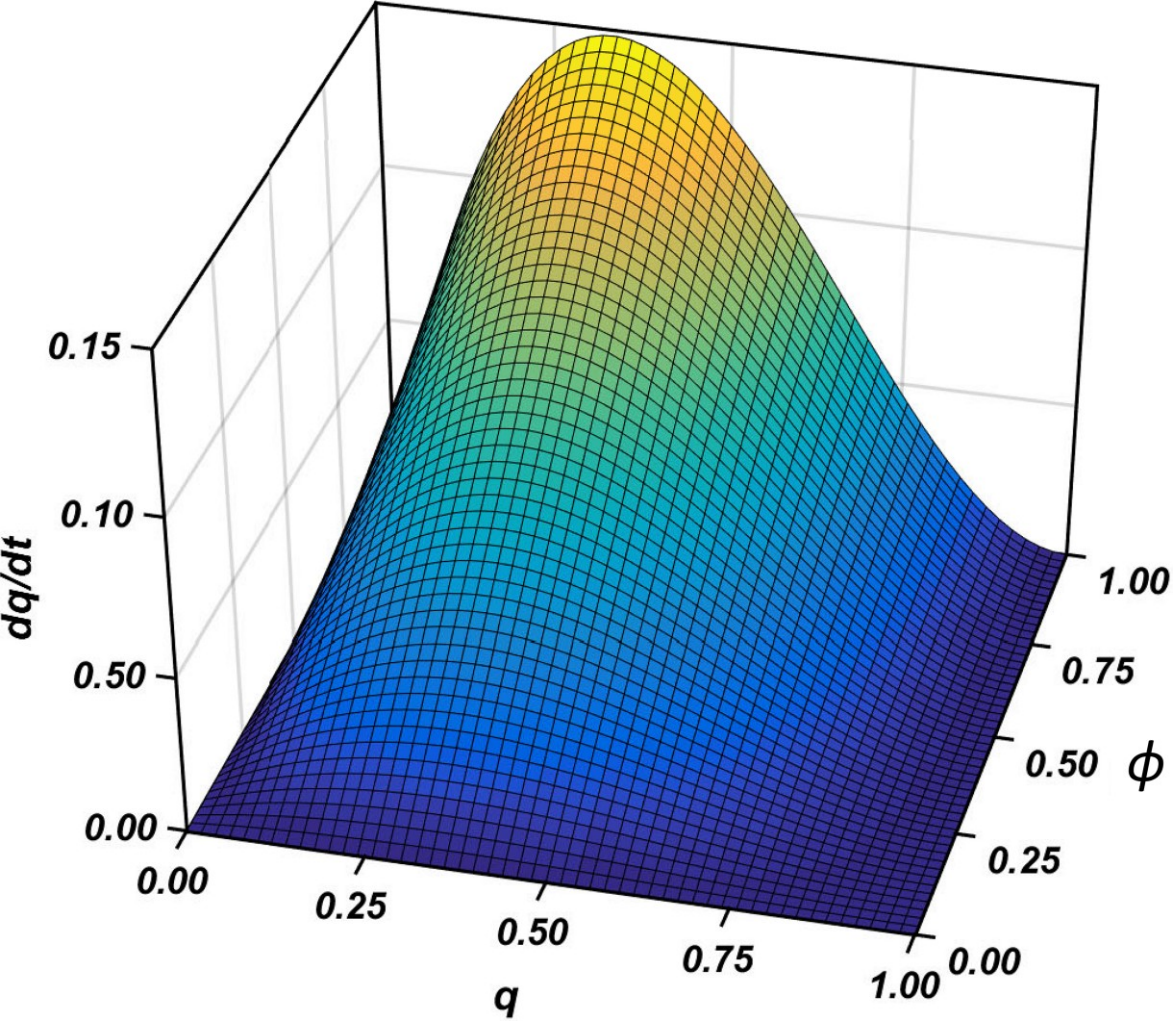
Replicator dynamics. Three-dimensional plot of the dynamic of the antibiotic prescribing game determined by the replicator equation. The *ϕ* axis represents the probability that a symptomatic patient has a bacterial infection, and the *q* axis represents the proportion of symptomatic patients in the population receiving antibacterial medication. The vertical axis is the value of $\dot{q}$ = *dq/dt*.

Fig 2 illustrates the resulting replicator dynamics for values of *ϕ* ∈ (0, 1] and *q* ∈ [0, 1]. Vectors $\dot{q}$ are positive except at *q* = 0 and *q* = 1. The lengths of the vectors (height) represent rates of increase in antibiotic prescribing. Antibiotic prescribing increases most rapidly when *ϕ* (the probability that a symptomatic patient has a bacterial infection) approaches 1 and when *q* (the proportion of patients receiving antibiotics) is 1/3. The state *q* = 1, with all patients receiving antibiotic treatment, is a strict Nash equilibrium and is *asymptotically stable* and a *global attractor*, meaning not only that it is an evolutionarily stable Nash equilibrium, but also that there is a dynamic attraction from all other states to the outcome in which all symptomatic patients receive antibiotic treatment. The state *q* = 0, where no symptomatic patients receive antibiotic treatment, is an unstable rest point and hence an unstable equilibrium.

**Fig 3:**
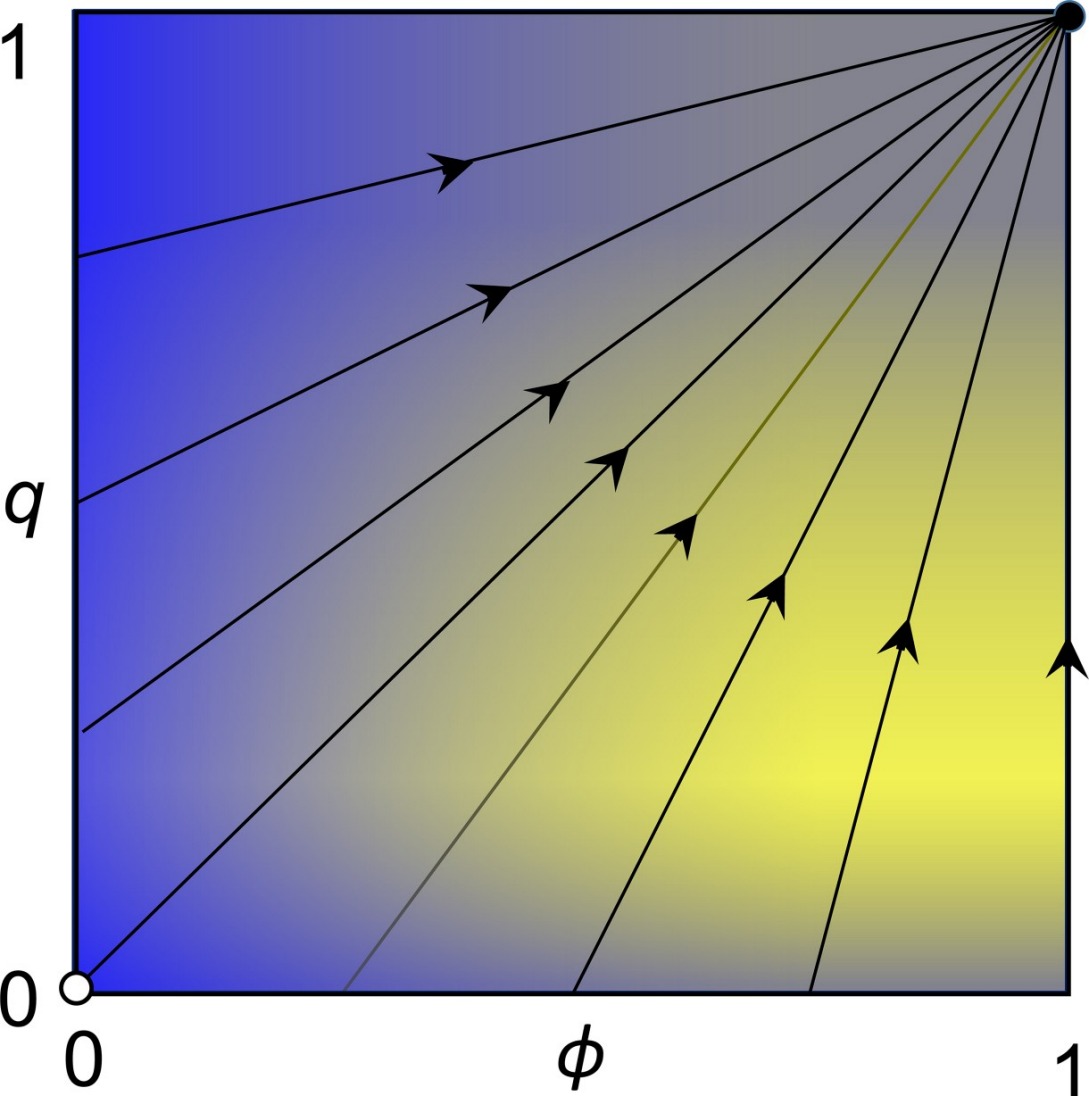
Replicator dynamics. The set of points determined by values of *ϕ* and *q*, illustrating the basin of attraction and typical trajectories of the replicator dynamics, with an unstable rest point where *ϕ* = *q* = 0 and stable rest point and global attractor at *ϕ* = *q* = 1. The shading indicates most rapid change at high values of *ϕ* and *q* = 1/3.

Fig 3 shows the set defined by all possible values of *ϕ* and *q*, typical trajectories determined by the replicator dynamics, an unstable rest point at *ϕ* = *q* = 0, and a global attractor at *ϕ* = *q* = 1, where antibiotic prescribing is maximized. Because T is a dominant strategy, the *basin of attraction* from which all trajectories converge on the global attractor includes all states in which *ϕ* > 0 and 0 < *q* < 1.

### Limitations of the model

Every mathematical model depends on simplifying assumptions, and our antibiotic prescribing game is no exception. It is worth drawing attention to a few of the assumptions in our own model to avoid overgeneralized interpretations or applications of the game. One important assumption, alluded to earlier, is that prescribing doctors are motivated solely to minimize morbidity and mortality in their own patients when they present for treatment. If doctors are instead motivated in some other way, for example, if they strive to maximize the welfare of all patients in the population, present and future, then the antibiotic prescribing game fails to provide an accurate model of their behavior.

Second, we assumed that, if all symptomatic patients are treated with antibiotics (if *q* = 1), then antibiotic resistance will have reduced the therapeutic efficacy of antibiotics to zero. However, it is possible to imagine a situation in which, for a limited time, antibiotics might retain their efficacy even while all symptomatic patients are receiving them. In those circumstances, a doctor’s payoff for choosing T (treat with antibiotics) decreases as *q* increases but remains larger than the payoff for U (do not treat with antibiotics) even when *q* = 1. Eq 7 is then replaced by *π*_*i*_(1,*q*) = −*λq*(0<*λ*<*ϕ*). The average payoff to a doctor in the game is then *π*_*i*_(*p*_*i*_,*q*) = *q*(−*λq*)−*ϕ*(1−*q*), and the payoff is maximized when *q* = 1, for *λ* < *ϕ*/2. However, it is immediately obvious from Fig 1 that T is then a strictly dominant strategy, because the T payoff function lies above the U payoff function for all values of *y*, and it follows that the outcome in which every doctor chooses T is a unique and stable Nash equilibrium, but the game is no longer a tragedy of the commons, because this outcome is not worse for every doctor than the outcome when every doctor chooses U or a mixed strategy *p*_*i*_.

It may also be possible for antibiotics to lose efficacy entirely before all symptomatic patients are medicated with them. In that scenario, the payoff of the T and U payoff functions meet at some value *q*′ < 1, and if antibiotic medication has any adverse side-effects affecting morbidity, then the T function crosses the U function, and for *q* > *q*′ the payoff for choosing U is greater than the payoff for choosing T. In these cases, the meeting or intersection point at *q*′ represents a Nash equilibrium, and the antibiotic prescribing game is not an entirely appropriate model.

### Conclusions

The fundamental theoretical implication of our analysis is that the strategic structure of the antibiotic prescribing game motivates payoff-maximizing doctors to prescribe antibiotics, irrespective of the extent of antibiotic resistance in the population and irrespective also of the probability (as long as it is nonzero) that symptomatic patients are infected with pathogenic bacteria. From any initial pattern of prescribing behavior, doctors will tend over time to increase antibiotic prescribing. This is true despite the fact that any more restrained prescribing strategy, apart from never prescribing antibiotics, yields a better payoff to every doctor, provided that all doctors choose it. The payoff to every doctor is maximized when antibiotics are prescribed to half the symptomatic patients in the population, in practice according to their judgments of the probability of severe bacterial infection. The strategy of prescribing antibiotics to all symptomatic patients may lead to the evolution of antibiotic resistance, ultimately rendering antibiotics useless for treating even the most dangerous infections and also reducing the doctors’ own payoffs. Thus, doctors’ decision making leads inexorably to an outcome that is worse for all of them and their patients than the outcome that would have resulted had they deviated from the game-theoretic rational strategy and exercised restraint. We have therefore proved that the antibiotic prescribing game is a social dilemma of the tragedy of the commons type.

This proof has significant implications for the design of interventions to reduce over-use of antibiotics in healthcare settings. The importance of drawing on theories of behavior in the design and implementation of antibiotic stewardship interventions is increasingly recognized [29], with evidence that interventions designed in line with psychological theories of behavior change are more likely to be effective [30]. We argue, however, that generic models of behavior change do not address the specific features of antibiotic prescribing as a social dilemma. Establishing that antibiotic prescribing in healthcare can be characterized as a social dilemma game gives weight to arguments raised by others that addressing the threat of resistance requires drawing on strategies for dealing with a tragedy of the commons [31–34].

Managing this social dilemma requires changing the payoffs of the game, and one way of achieving that is through coercive strategies that promote the good of society over individual interests, although there are both ethical and practical challenges to the restriction and control of antibiotics, particularly in the context of low-income countries where lack of access to antibiotics is a significant threat to health at a societal level [35]. We need to look to systems for the regulation, management, and monitoring of antibiotic prescribing, but evidence suggests that these systems may be best managed through cooperative, local community-based approaches [36–38] within which prescribers can agree mutual goals, build norms of cooperation, and use reputational incentives and sanctions to promote responsible antibiotic use. Interventions that seek to shift individual prescriber behavior by changing the payoff structure in other ways, for example by rewarding judicious antibiotic use, are also needed.

Changing behavior in the context of a tragedy of the commons problem is notoriously difficult. However, drawing on theory and evidence from the field of research into social dilemmas in a systematic way to inform intervention development will be critical as part of efforts to protect the antibiotic commons for the future. As Daniel Bernoulli commented in a paper presented to the French Academy of Sciences in 1760: “I simply wish that, in a matter which so closely concerns the wellbeing of the human race, no decision shall be made without all the knowledge which a little analysis and calculation can provide” [39].

## Appendix

### Conditions for evolutionary stability

Here we provide a proof of the well-known proposition that Inequalities 1 and 2, taken together, imply Inequality 3, and that Inequality 3 implies Inequalities 1 and 2. First, we prove that Inequality 1, when it holds strictly, implies Inequality 3.

According to Inequality 1, $\pi_{i}\left(p_{i}^{*},p_{-i}^{*}\right)\geq \pi \left(p_{i},p_{-i}^{*}\right)$ for all *p*_*i*_ ∈ *P*_*i*_. If this inequality is strict, and we let ϵ → 0 in Inequality 3, reproduced here as Inequality A1,

$$\pi_{i}\left(p_{i}^{*},\epsilon p_{-i}+\left(1-\epsilon \right)p_{-i}^{*}\right)>\pi_{i}\left(p_{i},\epsilon p_{-i}+\left(1-\epsilon \right)p_{-i}^{*}\right),$$ (A1)

then this inequality is satisfied for ϵ = 0 and for sufficiently small ϵ. This proves that Inequality 1, if it holds strictly, implies Inequality 3.

Suppose now that Inequality 1 holds with equality, so that

$$\pi_{i}\left(p_{i}^{*},p_{-i}^{*}\right)=\pi_{i}\left(p_{i},p_{-i}^{*}\right),$$

and that Inequality 2 holds, so that

$$\pi_{i}\left(p_{i}^{*},p_{-i}\right)>\pi_{i}\left(p_{i},p_{-i}\right).$$

We prove that Inequality 2 implies Inequality 3 as follows.

First, because *π*_*i*_ is continuous at 0, we can rewrite Inequality 3 as follows:

$$\epsilon \pi_{i}\left(p_{i}^{*},p_{-i}\right)+\left(1-\epsilon \right)\pi_{i}\left(p_{i}^{*},p_{-i}^{*}\right)>\epsilon \pi_{i}\left(p_{i},p_{-i}\right)+\left(1-\epsilon \right)\pi_{i}\left(p_{i},p_{-i}^{*}\right).$$ (A2)

We multiply both sides of $\pi_{i}\left(p_{i}^{*},p_{-i}\right)>\pi_{i}\left(p_{i},p_{-i}\right)$ by ϵ and, noting that $\pi_{i}\left(p_{i}^{*},p_{-i}^{*}\right)=\pi_{i}\left(p_{i},p_{-i}^{*}\right)$, we add $\left(1-\epsilon \right)\pi_{i}\left(p_{i}^{*},p_{-i}^{*}\right)$ to the left-hand side and $\left(1-\epsilon \right)\pi_{i}\left(p_{i},p_{-i}^{*}\right)$ to the right-hand side. This yields Inequality A2, and therefore Inequality 3, as required.

To prove that Inequality 3 implies Inequalities 1 and 2, taken together, we note first that if we examine the limit as ϵ → 0, then

$$\pi_{i}\left(p_{i}^{*},\epsilon p_{-i}+\left(1-\epsilon \right)p_{-i}^{*}\right)>\pi_{i}\left(p_{i},\epsilon p_{-i}+\left(1-\epsilon \right)p_{-i}^{*}\right)$$ (A3)

implies $\pi_{i}\left(p_{i}^{*},p_{-i}^{*}\right)\geq \pi_{i}\left(p_{i},p_{-i}^{*}\right),$ establishing Inequality 1.

Now suppose that

$$\pi_{i}\left(p_{i}^{*},p_{-i}^{*}\right)=\pi_{i}\left(p_{i},p_{-i}^{*}\right).$$

Then, from Inequality A2 we have $\epsilon \pi_{i}\left(p_{i}^{*},p_{-i}\right)>\epsilon \pi_{i}\left(p_{i},p_{-i}\right).$ It follows that

$$\pi_{i}\left(p_{i}^{*},p_{-i}\right)>\pi_{i}\left(p_{i},p_{-i}\right),$$

and this is Inequality 2, as required.

### Replicator equation

We assume that prescribing decisions are made repeatedly over time, and the replicators in this analysis are pure strategies T (treat with antibiotics) and U (do not treat with antibiotics). From Eqs 7 and 8, the payoff associated with the pure strategy T is *π*(1,*q*) = −*ϕq*, and the payoff associated with U is *π*(0,*q*) = −*ϕ*. The replicator equation is defined as the current relative frequency of T multiplied by the payoff associated with T relative to the average payoff from both strategies.

The average payoff from both strategies, when the current relative frequency is *q*, is given by

$$\pi \left(q\right)=q\left(-\phi q\right)+\left(1-q\right)\left(-\phi \right)=\phi \left(-q^{2}+q-1\right).$$ (A4)

We can now write the differential replicator equation

$$\dot{q}=\frac{dq}{dt}=q\left[-\phi q-\phi \left(-q^{2}+q+1\right)\right].$$ (A5)

Simplifying, we have

$$\dot{q}=\frac{dq}{dt}=\phi q{\left(1-q\right)}^{2},$$ (A6)

and this is Eq 9, as required.

## Supporting information

S1 Table (XLSX)
